# Acceptability of supervised injection facilities among persons who inject drugs in upstate New York

**DOI:** 10.1186/s12954-022-00665-x

**Published:** 2022-07-30

**Authors:** Eliana Duncan, Sarah Shufelt, Meredith Barranco, Tomoko Udo

**Affiliations:** 1 Department of Epidemiology and Biostatistics, School of Public Health, University at Albany, 1 University Place, Rensselaer, NY 12144 USA; 2 Department of Health Policy, Management, and Behavior, School of Public Health, University at Albany, 1 University Place, Rensselaer, NY 12144 USA; 3 Center for Collaborative HIV Research in Practice and Policy, School of Public Health, University at Albany, 1 University Place, Rensselaer, NY 12144 USA

**Keywords:** Supervised injection facilities, Persons who inject drugs, Drug overdose, Harm reduction, New York State

## Abstract

**Background:**

Supervised injection facilities (SIFs) provide spaces where persons who inject drugs (PWID) can inject under medical supervision and access harm reduction services. Though SIFs are not currently sanctioned in most of the US, such facilities are being considered for approval in several Upstate New York communities. No data exist from PWID in Upstate New York, and little from outside major US urban centers, on willingness to use SIFs and associated factors.

**Methods:**

This analysis included 285 PWID (mean age = 38.7; 57.7% male; 72.3% non-Hispanic white) recruited for a study on hepatitis C prevalence among PWID in Upstate New York, where participants were recruited from syringe exchange programs (*n* = 80) and able to refer other PWID from their injection networks (*n* = 223). Participants completed an electronic questionnaire that included a brief description of SIFs and assessed willingness to use SIFs. We compared sociodemographic characteristics, drug use/harm reduction history, healthcare experience, and stigma between participants who were willing vs. unwilling to use such programs.

**Results:**

Overall, 67.4% were willing to use SIFs, 18.3% unwilling, and 14.4% unsure. Among those reporting being willing or unwilling, we found higher willingness among those who were currently homeless (91.8% vs. 74.6%; *p* = 0.004), who had interacted with police in the past 12 months (85.7% vs. 74.5%; *p* = 0.04), and who were refused service within a healthcare setting (100% vs. 77.1%; *p* = 0.03).

**Conclusion:**

Our results support SIF acceptability in several Upstate New York PWID communities, particularly among those reporting feelings of marginalization. A large proportion reported being unsure about usage of SIFs, suggesting room for educating PWID on the potential benefits of this service. Our results support SIF acceptability in Upstate New York and may inform programming for underserved PWID, should SIFs become available.

## Background

Within the continuum of services for persons who inject drugs (PWID), there is an ongoing need for open minded, stigma-free medical programming in order to address adverse health outcomes associated with drug use, particularly overdose [1, 2]. Supervised injection facilities (SIFs) provide safe, low-barrier settings in which PWID may inject under the supervision of trained staff, as well as access services that reduce drug-related harm such as safe injection education, naloxone and overdose prevention, fentanyl test strips, and sterile injection equipment [3, 4]. Furthermore, SIFs may provide referral services to clients interested in detox and substance use disorder treatment programs, as well as referral to other social, legal, and medical services [3].

The efficacy and safety of SIFs are supported by growing scientific evidence, particularly in international contexts. Since the first drug consumption site opened in Switzerland in 1986, additional facilities have become operational in countries including Australia, Canada, the Netherlands, Germany, Spain, and Norway [5]. Since establishment of these programs, numerous independent studies have demonstrated SIFs to be associated with decreases in overdose morbidity and mortality, decreases in HIV and injection-associated infections, enrollment in detoxification and rehabilitation services, decreases in outdoor injecting, reduction in equipment sharing, and delays in average onset of injection drug use [6–11]. In addition, multiple studies have observed community benefits related to the presence of SIFs, including decreases in criminal incidents related to drug use, decreases in publicly discarded injection equipment, decreases in average usage of ambulatory services, as well as overall cost-effectiveness [6, 12].

Aside from research demonstrating the benefits of SIFs, studies evaluating acceptance and perceptions of SIFs have been conducted among multiple stakeholder groups. Feasibility studies, particularly in Canadian contexts, have found that PWID commonly felt that SIFs increased their physical safety (i.e. decreased exposure to police interaction and criminal activity) and reduced risks associated with drug use (i.e. public injecting associated with rushed and unhygienic injecting practices) [12, 13]. In the US, varying degrees of acceptance from policymakers and community members exist regarding SIFs. Some research in the US context has assessed acceptability of such facilities among PWID in urban areas such as Philadelphia, Baltimore, Providence, and San Francisco, finding an overwhelming majority of PWID willing to use SIFs [14–17]. Though limited data exist on unsanctioned SIFs and their clientele, existing US studies focusing on anonymous, unsanctioned sites have observed multiple benefits, primarily related to their ability to access PWID who may typically engage in high-risk injecting practices [18, 19]. Legally sanctioned SIFs are largely nonexistent in the US, however such facilities have recently been piloted in New York City, and have been proposed in several New York State (NYS) locations. With calls for SIF approval at a state level, there is an urgent need to understand acceptability of such facilities by PWID in Upstate New York communities [20]. Such data is particularly vital given increases in fatal and non-fatal overdoses driven by the COVID-19 pandemic, as well as increasing fentanyl-related morbidity and mortality [21]. This analysis aims to assess the acceptance of SIFs among PWID in Upstate New York and explore specific subgroups that may most benefit from such facilities.

## Methods

### Participants

Participants were drawn from the Upstate PWID Study for Infectious Disease Elimination (UPSIDE), a larger study that aimed to estimate the prevalence of Hepatitis C Virus (HCV) and Human Immunodeficiency Virus (HIV) infection among PWID in Upstate New York, investigate associated behavioral and social risk factors, and assess facilitators and barriers to accessing HCV and HIV care. Participants were recruited from three syringe exchange programs (SEPs) in Albany, Plattsburgh, and Norwich. Respondent-driven sampling was employed to recruit additional participants, where seed participants were asked to refer up to three peers who were also current PWID. Participants received up to $50 in gift cards for their time to complete the study procedures and $20 per referral of a peer, up to three peers. Study eligibility included those who were aged eighteen years and older, had injected drugs in the previous 12 months, were able to read and write in English, resided in selected counties that the collaborating SEPs generally served, and had no immediate plans for relocation. Participants who agreed and were eligible to participate completed a self-administered questionnaire, either on-site or on their own electronic devices. UPSIDE protocol was approved by University at Albany Institutional Review Board.

### Measures

Participants were given a brief description of SIFs and told that none were available in NYS. They were then asked the question, “If one was available, would you use such a safe injection facility?” with response options, 1 = Yes, 0 = No, and 9 = Don’t know/not sure. Participants also answered questions about sociodemographic characteristics, drug use history, harm reduction behavior, sexual risk, healthcare-seeking behavior and experiences, experienced and perceived stigma, and mental health status.

### Analysis

Descriptive statistics were used to examine the distribution of characteristics among our analytic sample. Chi-square and Fisher’s exact tests were run in order to compare sociodemographic characteristics and drug use behaviors by acceptability of SIFs, at a significance level of *p* < 0.05. All analyses were completed using SAS 9.4 software [22].

## Results

This analysis included 285 PWID recruited for a study to assess the prevalence of hepatitis C among PWID in Upstate New York, with 80 participants recruited from SEP visits and 223 referrals recruited from their injection networks (Table 1). This study excluded 18 participants who were part of the larger UPSIDE study, but did not answer the SIF question. Our sample was majority male (57.7%) and non-Hispanic white (72.3%), with a mean age of 38.7 years (SD = 10.8). A majority of our sample had at least a high school education (77.2%) and 26.1% of participants identified as homeless or unstably housed. Regarding drug use history and injecting behavior, 50.2% our sample injected daily, 79.3% most often injected in private, and 44.6% had ever overdosed.

**Table 1:** Selected characteristics of PWID assessed for willingness (no vs. yes) to use SIFs (*n* = 244)

Variable	No		Yes		*p*-value
	*n* = 52	(%)	*n* = 192	(%)	
Gender					
Male	27	(18.8)	117	(81.3)	0.42
Female	25	(25.3)	74	(74.8)	
Non-binary	0	(0)	1	(100.0)	
Education					
Less than high school	10	(17.5)	47	(82.5)	0.43
At least high school/GED	42	(22.5)	145	(77.5)	
Currently homeless					
No	46	(25.4)	135	(74.6)	0.004
Yes	5	(8.2)	56	(91.8)	
Drugs injected at least once in past 30 days					
No	28	(26.4)	78	(73.6)	0.09
Yes	23	(17.3)	110	(82.7)	
Injections per day					
1–2	22	(27.2)	59	(72.8)	0.07
3–4	20	(22.5)	69	(77.5)	
> 5	8	(11.9)	59	(88.1)	
Primary injecting location					
Private	42	(21.7)	152	(78.4)	0.66
Public	9	(18.8)	39	(81.3)	
Ever overdose					
No	28	(21.7)	101	(78.3)	0.93
Yes	24	(21.2)	89	(78.8)	
Visited SEP (past 12 mo)					
No	26	(22.8)	88	(77.2)	0.78
Yes	21	(21.2)	78	(78.8)	
Ever used narcan					
No	34	(21.8)	122	(78.2)	0.62
Yes	15	(19.0)	64	(81.0)	
Interaction with police (past 12 mo)					
No	38	(25.5)	111	(74.5)	0.04
Yes	13	(14.3)	78	(85.7)	
Ever arrested or incarcerated (past 12 mo)					
No	16	(26.2)	45	(73.8)	0.26
Yes	35	(19.4)	145	(80.6)	
Given less attention than other patients in a healthcare setting					
No	47	(23.9)	150	(76.1)	0.05
Yes	5	(10.6)	42	(89.4)	
Treated with hostility by a healthcare provider					
No	44	(22.8)	149	(77.2)	0.27
Yes	8	(15.7)	43	(84.3)	
Refused service by a healthcare provider					
No	52	(22.9)	175	(77.1)	0.03
Yes	0	(0)	17	(100)	

Excludes those who were unsure about their acceptance of SIFs

Overall, 67.4% (*n* = 192) of our analytic sample reported willingness to use SIFs, 18.3% (*n* = 52) reported being unwilling, and 14.4% (*n* = 41) reported being unsure of use. Among those reporting either being willing or unwilling, the proportion of PWID reporting willingness to use SIFs was greater among those who were currently homeless compared to those who were not currently homeless (91.8% vs. 74.6%; *p* = 0.004), who had interacted with police in the past 12 months compared to those who had not had such interactions (85.7% vs. 74.5%; *p* = 0.04), and who had been refused service within a healthcare setting compared to those who had not been refused service (100% vs. 77.1%; *p* = 0.03).

## Discussion

Our analysis points to strong acceptability of and willingness to use SIFs among several Upstate New York PWID communities. We also found significant associations between acceptance of SIFs and indicators of greater social need and marginalization within society, such as current homelessness, interaction with police in the past twelve months, and being refused service within a healthcare setting.

Such results align with existing research. First, homelessness has been associated with negative aspects of drug use, such as increased risk of overdose, increased intensity of drug use, increased risk of relapse when in recovery, and greater participation in risky income-generating activity [23–25]. To this point, homeless PWID are often in need of more comprehensive services than those who are stably housed, and SIFs may be able to fulfill this need through referrals [23]. Furthermore, PWID commonly have negative interactions with law enforcement, and those who are homeless or living transiently are particularly at risk for police scrutiny [26, 27]. In line with existing literature, our results may point to SIFs having the potential to not only provide health benefits, but also act as safe spaces against discrimination experienced through over policing and place-based policing of PWID spaces [28].

In terms of treatment within healthcare settings, our results fall in line with existing literature on PWID experiences around healthcare utilization. PWID frequently avoid medical services when needed due to perceived and actual stigma from healthcare professionals, and this may result in worse health outcomes and need for eventual acute or emergency care [29, 30]. This highlights the need for non-judgmental, friendly medical services for PWID and may explain the greater acceptability of SIFs by those who are either given less attention in medical settings or refused medical services within our analytic sample.

To our knowledge, this analysis is the first to date assessing willingness of PWID to use SIFs in Upstate New York. Our study may be restricted by a few limitations. For instance, we sampled a hard-to-reach population, and given the lack of an available sampling frame, we may not have penetrated all subgroups of PWID within the larger community. Furthermore, we collected potentially sensitive information from study participants, and this may have introduced bias in data collection. However, measures were taken to mitigate concerns around confidentiality and trust, such as self-administration of the questionnaire and utilization of a peer recruitment strategy. Lastly, statistical power may be an issue for our findings, given this was a secondary, exploratory analysis.

Our results also suggest a few directions for future research on SIFs. For instance, a notable proportion of our sample reported being unsure about or unwilling to use SIFs. Given PWID do not universally support or adopt harm reduction interventions, it will be important to further explore factors that may influence their use, such as geographical setting, legality and political climate, and cultural norms. Understanding such nuances underlying responses among our sample may be warranted in order to establish effective interventions for SIF-hesitant populations. Finally, our results support SIF acceptability in Upstate New York PWID communities as a whole, and emphasize the need to provide comprehensive care to marginalized subpopulations. While this is an important observation, should SIFs become operationalized in communities outside of New York City, ongoing programmatic evaluation will be necessary in order to build on these findings and better understand SIF utilization patterns in practice, especially in light of the immediate and lasting effects of the COVID-19 pandemic on PWID populations.

## Data Availability

The dataset analyzed during the current study is not publicly available in order to protect study participant privacy.

## Abbreviations

HIV: Human immunodeficiency virus
HCV: Hepatitis C virus
NYS: New York state
PWID: Persons who inject drugs
SIF: Supervised injection facilities
